# Visual-, Olfactory-, and Nectar-Taste-Based Flower Aposematism †

**DOI:** 10.3390/plants13030391

**Published:** 2024-01-29

**Authors:** Simcha Lev-Yadun

**Affiliations:** Department of Biology & Environment, Faculty of Natural Sciences, University of Haifa—Oranim, Tivon 36006, Israel; levyadun@research.haifa.ac.il

**Keywords:** aposematic coloration, flower, herbivory, olfactory, poisonous plants, secondary metabolites, toxic nectar

## Abstract

Florivory, i.e., flower herbivory, of various types is common and can strongly reduce plant fitness. Flowers suffer two very different types of herbivory: (1) the classic herbivory of consuming tissues and (2) nectar theft. Unlike the non-reversibility of consumed tissues, nectar theft, while potentially reducing a plant’s fitness by lowering its attraction to pollinators, can, in various cases, be fixed quickly by the production of additional nectar. Therefore, various mechanisms to avoid or reduce florivory have evolved. Here, I focus on one of the flowers’ defensive mechanisms, aposematism, i.e., warning signaling to avoid or at least reduce herbivory via the repelling of herbivores. While plant aposematism of various types was almost ignored until the year 2000, it is a common anti-herbivory defense mechanism in many plant taxa, operating visually, olfactorily, and, in the case of nectar, via a bitter taste. Flower aposematism has received only very little focused attention as such, and many of the relevant publications that actually demonstrated herbivore repellence and avoidance learning following flower signaling did not refer to repellence as aposematism. Here, I review what is known concerning visual-, olfactory-, and nectar-taste-based flower aposematism, including some relevant cases of mimicry, and suggest some lines for future research.

## 1. Introduction

Colorful flowers are the most common and best-known visual characteristic used by plants to communicate with animals. The most common and most studied aspect of this visual communication of flowers with potential pollinators is their attraction (e.g., [1,2,3,4,5,6,7]). Another specific aspect of color-related visual flower–pollinator interactions is a change in flower color as a signal, which shows that specific flowers are already pollinated [8,9,10]. In parallel to common visual signaling, many flowers attract pollinators (and herbivores) via olfactory signals and cues (e.g., [1,4,11]). Flower colors may also serve to regulate their temperature [12,13,14,15]; as such, they not only help with gamete development in cold environments (e.g., [16,17]) but even provide a reward for pollinators in habitats or during hours characterized by low temperatures (e.g., [6,14,18,19]). It should be remembered that excess sunlight, especially in the UV (ultraviolet) spectrum, may cause damage to reproductive mechanisms, especially gametes [17,20]; indeed, sunlight was found to influence flower color polymorphism in *Lysimachia arvensis* [21] and in many other species [22]. Along with pollination and herbivory, physical environmental stresses also drive flower color evolution [12,13,15,23,24]. In spite of the various specific functions of flower coloration, flower color is, in many cases, the outcome of the pleiotropic effects of vegetative pigmentation [25]. As flowers are often nutritious, they are prone to high levels of florivory, i.e., flower herbivory, and they have evolved various mechanisms as defenses against herbivores [26]. I refer to nectar thieves as herbivores, as I consider them to be exactly the same as all animals that consume parts of flowers or whole flowers. Nectar theft is known as floral larceny [27].

In addition to pollinator attraction and the overcoming of various abiotic stresses, flower colors, shapes, nectar tastes, and odors also emerge as mechanisms involved in their defense against herbivores (e.g., [23,27,28,29,30]). Flower redness also seems to defend against fungal attacks in certain cases [31,32]; this is a characteristic that is also true for the redness of leaves [33,34,35] and fruit [36].

When discussing flower color, it is necessary to consider the fact that the way we see color is, in many cases, different from the color vision of both pollinators and herbivores. Many pollinating insects and birds see ultraviolet (UV) light, while humans do not, and many flowers signal to pollinators in the UV range (e.g., [37,38,39]). Moreover, the same colors can differ in their chromatic versus achromatic characteristics, and fruit expressed redder hues (e.g., [40]).

With only a few exceptions, aposematism (warning signaling) was hardly ever considered to be an anti-herbivory mechanism by botanists before the 21st century. Before the year 2000, the few papers that discussed the operation of visual aposematism in plants were about the poisonous ones (e.g., [38,41]). Harper ([42], p. 416) commented on the reluctance of botanists to accept plant aposematism: “botanists have been reluctant to accept precisions of adaptations that are commonplace to zoologists and often seem reluctant to see the animal as a powerful selective force in plant evolution except in the curiously acceptable realm of adaptation to pollination! It may be that much of the fantastic variation in leaf form, variegation, dissection and marking that is known in the plant kingdom is accounted for by the selective advantage to the plant of associating unpalatability with a visual symbol”. It should be remembered that being conspicuous for both visual and chemical signaling to attract potential pollinators exposes the conspicuous flowers to herbivory; thus, there is a conflict of interest between the conspicuousness that serves pollination and the need for better defenses following the increased risk of herbivory because of it.

The global decline in botanical education (e.g., [43]) requires a detailed introduction in order to serve many of the younger readers who commonly lack a broad and sound botanical education. Therefore, I provide a detailed theoretical background below. Here, I review the issues of visual-, olfactory-, and nectar-taste-based anti-herbivory defenses as repellence and deterrence (aposematism) in flowers.

### Aposematism

Aposematic signaling is a common biological phenomenon in which poisonous, spiny, dangerous, or otherwise unpalatable or unprofitable organisms advertise their defensive qualities to other organisms. This signaling serves as protection in plants against herbivores or as a defense in animals against carnivores. Aposematic signaling may be conveyed by color, movement, morphology, odor, taste, and even by sound [44,45]. As a rule, aposematism signals related to defense or unpalatability function from a lower trophic level to a higher one [44,46], including via chemical signaling between certain host plants towards specific parasitic plants [47]. The evolution of aposematic signaling is based on the ability of target enemies to associate the signaling with risk, damage, or non-profitable handling and, later, to avoid such organisms as prey or hosts [30,34,44,46,48,49,50,51]. The typical colors of aposematic animals are yellow, orange, red, purple, black, white, brown, and their specific combinations [44,46,48,52,53].

Aposematic coloration is expressed by many thorny, spiny, prickly, and poisonous plants and by plants that are unpalatable or of low nutritive value for various other reasons. Like it is in animals, aposematic coloration in plants is commonly yellow, orange, red, brown, black, or white, or the coloration involves specific combinations of these colors [30,34,41,49,50,54,55]. Moreover, plants can be fully or partly aposematic by acquiring, in different ways, defense traits and/or signaling from other organisms, including from other plants [56], fungi [57,58], bacteria [59], and insects (e.g., [60]).

It is critical to understand and consider the fact that many types of aposematic and other types of defensive plant coloration may simultaneously serve other functions, such as physiological and communicative functions and even other defensive functions (e.g., [5,30,34,35,50,61,62,63,64]). It is therefore difficult in many cases to evaluate the relative functional share of visual and chemical aposematism in various plant color or odor patterns and to identify the specific selective agents that were involved in their evolution [30,34]. Moreover, because of the extinctions following the drastic climatic changes during the Pleistocene and the current huge impact of human activity, it is probable that certain selection agents disappeared, leaving “orphaned” adaptations (see [34,65,66]).

I wish to stress and illuminate an issue commonly overlooked or ignored by many: in the vast majority of species of all the animal and plant taxa proposed or considered to be aposematic, aposematism has never been proven via the demonstration of avoidance learning or genetically based avoidance by their enemies. Despite this, the concept of aposematism is an excellent research tool that explains the many variable interactions among organisms, and it seems to explain, even if not exclusively, the existence of many characteristics in numerous animal and plant taxa.

Lev-Yadun [34] suggested considering defensive yellow and red autumn leaf coloration, to add a basic assumption when studying the anti-herbivory roles of such plant coloration; this is a principle that is fully relevant to flower aposematism. If a leaf or a flower characteristic deters various herbivores, it is not only the herbivore species that attack the plants that should be studied, as is almost always the case; it is also necessary to consider and test the other relevant herbivore taxa occurring in the same geography or ecology that do not attack the plants. Lev-Yadun [34] suggested that since the herbivore species that attack the plants are obviously fully or partly resistant to the various plant’s defenses, the herbivore species that occupy the same habitats as the plant species but do not attack should also be considered; this is because they may be the ones that are fully repelled and are therefore considered to not have interactions with the relevant plant species and thus they are always overlooked. Many previous studies and hot debates about defensive plant (and animal) coloration and odors missed that critical point and should possibly be re-evaluated.

Visually based aposematism is not the only common way to achieve herbivore repellence. A parallel and sometimes simultaneous way is by olfactory aposematism. Olfactory aposematism, whereby poisonous plants repel or deter mammalian or insect herbivores by volatiles (with or without simultaneous visual aposematism) was for many years studied and discussed more than visual plant aposematism (e.g., [60,67,68,69,70,71,72,73]), although many of the cases that were described and tested experimentally were not classified by the authors as aposematic; rather, they were classified only as cases of repellence. It is currently still unknown whether the aposematic signaling via color and odor is aimed toward the same herbivores or toward different receivers.

Defense by aposematic signaling is a frequent occurrence. One of the best proofs of its function, which already existed in Darwin’s days, is that it resulted in the evolution of many mimicking animals and plants (e.g., [30,44,46,48,50,51,53,74,75,76,77,78,79]). The evolution of mimicry requires models, mimics, and predators or herbivores (operators), which select for the mimicking phenotype. The model should be another species or a group of species, or it should be based on their actions (e.g., release of chemicals, visual aposematism, or physical damage caused to other organisms) [53,58]; however, it can be the same species and even an organ of the same individual [30,76,80].

Aposematic mimics are organisms that have evolved to resemble other aposematic organisms, focusing mainly on visual, olfactory, auditory, and physical characteristics. The aposematic mimics belong to two general categories: Müllerian and Batesian. Müllerian mimicry describes a phenomenon in which two or more species with effective defenses against predators and herbivores share similar appearances or signals. The shared signaling results in the sharing of the cost for the signaling species of associative learning by their enemies [75]. It reduces the damage inflicted on both the signaling defenders and their enemies if they refrain from attacking. Potentially, it may promote the evolution of a genetically based predator that refrains from attacks [30,44,46,50,51,53]. Batesian mimicry is a phenomenon in which members of a palatable species or a group of such species gain protection against predation or herbivory by resembling or mimicking the defensive signaling of a defended species or of a group of defended species [30,34,44,46,50,51,52,53,58,74,77,78,79,81,82]. However, there are intermediate situations known as quasi-Batesian mimicry, i.e., defended and signaling species that differ in terms of the strength of their defense or signaling [83].

The Müllerian and Batesian mimicry types were originally defined in defensive (anti-predatory) animal systems. Later, these terms were adopted by botanists studying pollination biology, which defined rewarding flowers as Müllerian mimics and non-rewarding flowers as Batesian mimics (e.g., [6,84]). The use of these terms in relation to pollination predated by many decades the much later understanding of how common defensive plant aposematism is, as well as the understanding of the common existence of defensive Müllerian and Batesian mimicry types in plants. Using the terms Müllerian and Batesian mimicry in a non-defensive context for rewarding/non-rewarding flowers is, however, confusing if not misleading, and it is also logically inappropriate. Following the accumulating evidence for the common occurrence of defensive Müllerian and Batesian mimicry types in plants, Lev-Yadun [85] suggested the following: first, stop using the terms Batesian and Müllerian mimicry in relation to rewarding/non-rewarding flowers and pollination; second, name the guild of flowers that reward pollinators as Darwinian mimics and those that do not reward pollinators as Wallacian mimics in order to honor these great scientists.

Concerning aposematism, Lev-Yadun [30] discussed and posited that no plant defense system is perfect and that defenses are relative; this is an issue that was already stated long ago with regard to various types of defenses [60,86,87]. The assumption that a perfect defense system could exist is naïve. All types of defenses probably have a cost, although the cost can be mitigated by the multifunctionality of some of the defenses and following several types of non-defensive gains by these characteristics, e.g., the physiological ones [30,34,61,64]. However, the best plant defense is to avoid attacks rather than to resist them or to regenerate after damage [50]. Aposematism, camouflage, mimicry, masquerade [30,49,51,82,88,89,90], and association with other organisms (e.g., [91]), are the common tactics by which plants avoid attacks.

Below, I discuss several types of repellence and deterrence of herbivores by flowers.

## 2. Flower Aposematism

There are several types of flower aposematism. Some of these types overlap or operate simultaneously to a certain extent; some serve in attracting certain pollinators while repelling herbivores or non-efficient pollinators; and some may also have physiological functions: (1) toxic plants, (2) spiny plants, (3) functions related to flower symmetry, (4) toxic or bitter nectar, (5) visual mimicking of dangerous animals, and (6) mimicry of odors of risks of various types that attract certain pollinators but may repel herbivores.

It is important to distinguish aposematism, a repelling or deterring strategy, from pollination efficiency-related selection, such as the “bee avoidance hypothesis”, where plants evolved to discourage bees and attract more efficient pollinators (e.g., [92,93]). Similarly, the scaring away of pollinators after they are loaded with pollen by the quick movement of stamens in order to enhance pollen dispersal [94,95] should also not be considered aposematism.

### 2.1. Aposematic Coloration in Toxic Flowers

Flower characteristics such as color and morphology and traits such as chemical and physical defenses are more often discussed in the context of mechanisms for filtering non-legitimate or less functional pollinators, such as in the context of pollinating birds versus insects (e.g., [1,5,9,96,97]), rather than being viewed as anti-herbivory aposematism. I start the discussion with the aposematism of toxic flowers because this is the first type of flower aposematism discussed in modern biology [28].

**Figure 1:**
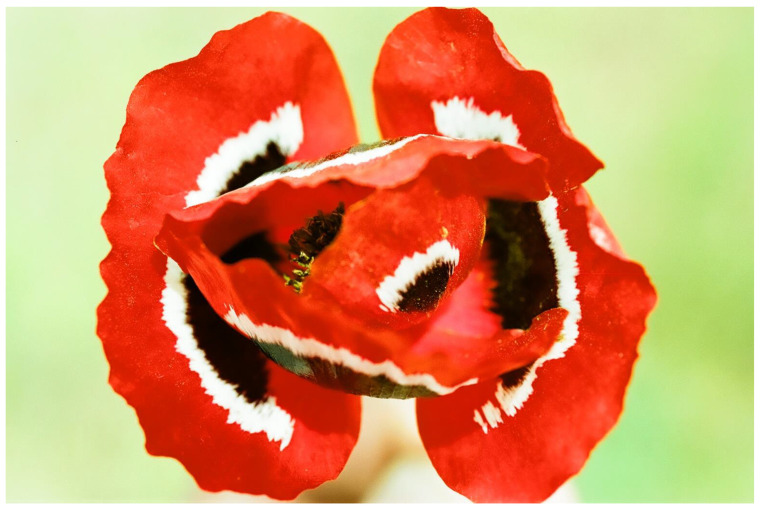
The strong contrasting red, white, and black colors of a large toxic and aposematic flower of *Papaver umbonatum* (Papaveraceae) from Mount Odem, the Golan Heights, Israel.

Half a century ago, the zoologist Hinton [28] proposed that yellow, red, and other types of vivid flower coloration of poisonous flowers should not only be considered attractive for pollinators; colorful poisonous flowers should also be considered aposematic and as a defense against herbivory (Figure 1).

He also proposed that the visually aposematic flowers probably have mimics. This was one of the first proposed cases of plant aposematism. It was published as a chapter in a book about illusion, aimed mostly at artists and not biologists, and I had a copy of that book because at that time I was still a professional photographer. This hypothesis was briefly mentioned by the very influential natural scientist Dame Miriam Louisa Rothschild [54] when she discussed the various roles of carotenoids in plants and animals, but for many years, this hypothesis regarding chemically based visual floral aposematism was not discussed. For instance, two decades later, Lamont [98] proposed that in red-flowered cyanogenic *Grevillea* species, the red color may be a cue for higher animals to be aware of their poisonous nature. Lamont’s proposal did not refer to aposematism, and of course, a cue is not a signal. However, I think that in this case it is indeed a classic visual aposematic signal, the first one proposed to be operating in the flowers of a specific toxic plant taxon.

The metabolic association of conspicuous flower color with chemical defense was discussed by Fineblum and Rausher [99], but without mentioning aposematism. The biochemical basis for this correlation is that anthocyanins and a number of defense chemicals, such as tannins, stem from the same biosynthetic pathways. Lev-Yadun and Gould [62,63], in a study of colorful autumn leaves, and Lev-Yadun [100], in a study of all plant organs, proposed that the association of conspicuous coloration with defensive chemicals should be considered aposematic. Irwin et al. [101] and Strauss and Irwin [102] showed that the red flower morphs of the wild radish *Raphanus sativus*, which were also richer in secondary defensive metabolites than white morphs, were better defended against various herbivores. These results were later corroborated by both generalist and specialist Brassicaceae herbivores that caused significantly more floral damage to white-flowered types than to pink-flowered ones [103]. Hanley et al. [104] proposed that the red cyanogenic flowers of several Australian *Hakea* species deter florivores. Tsuchimatsu et al. [105] showed that the white-flowered types of *Geranium thunbergia* were attacked more by the weevil *Zacladus geranii* than the pink-flowered ones. While Irwin et al. [101], Strauss and Irwin [102], Strauss and Whittall [23], Hanley et al. [104], McCall et al. [103], and Tsuchimatsu et al. [105] did not use the term aposematic, they actually described its operation with regard to different flower color types. In accordance with the above, Vaidya et al. [106], without mentioning aposematism, found that plants of the purple-flowered morph of *Boechera stricta* experienced lower foliar herbivory than plants of the white-flowered morph.

Gerchman et al. [107] tested the question of whether visual signaling for pollinators can simultaneously serve aposematism. They used as their experimental system the conspicuous purple tufts of leaves (“flags”), which often terminate the vertical inflorescences of the Mediterranean annual *Salvia viridis*. These flags were already shown to attract insect pollinators to the flowering patch [108]. Gerchman et al. [107] determined the aposematic potential of *S*. *viridis* flags on three levels: (1) the concentrations of anthocyanins, which, it is suggested, function as the visual aposematic signal in flags versus leaves; (2) the use of spectrometry to estimate whether the color vision system of two common but very different Mediterranean generalist herbivores (locusts and goats) can discriminate colorful flags from green leaves; and (3) the performance of feeding choice experiments to determine the food preferences of these herbivores. The anthocyanin concentrations in the flags were found to be more than 10 times higher than those in the leaves. The flags exhibited peak reflectance at 450 and 700 nm wavelengths, while the leaves reflected maximally at 550 nm. The goats preferred feeding on clipped inflorescences over intact control inflorescences. The locusts preferred consuming green leaves over colorful flags. To test whether this was due to deterrence from the flags’ coloration, the authors also offered them a choice between leaves and a rare, white morph of the flags. The locusts chose both equally immediately after presentation, but the leaves attracted more individuals after five minutes of feeding. The locusts also preferred green cabbage over anthocyanin-rich red cabbage. The author’s results supported the possibility of a secondary function of colorful extra-floral displays as a defensive warning signal.

### 2.2. Flower Aposematism in Spiny Plants

**Figure 2:**
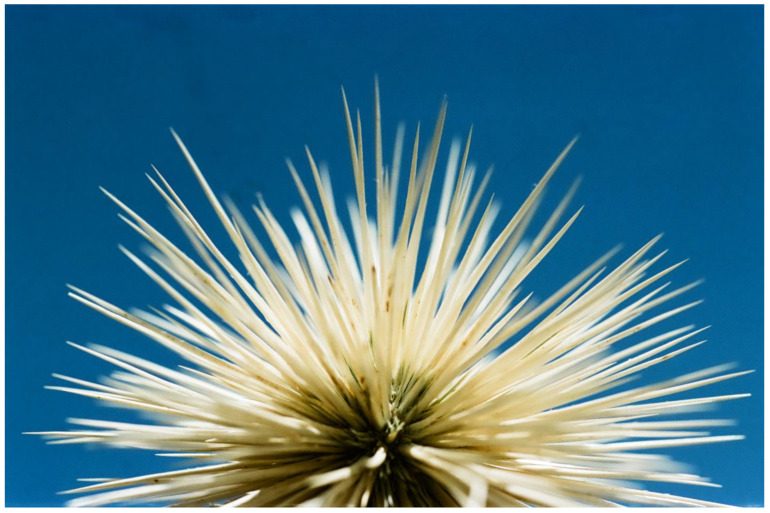
The light-colored and highly spiny aposematic inflorescence of the desert species *Echinops polyceras* (Asteraceae), central Negev Desert, Israel.

**Figure 3:**
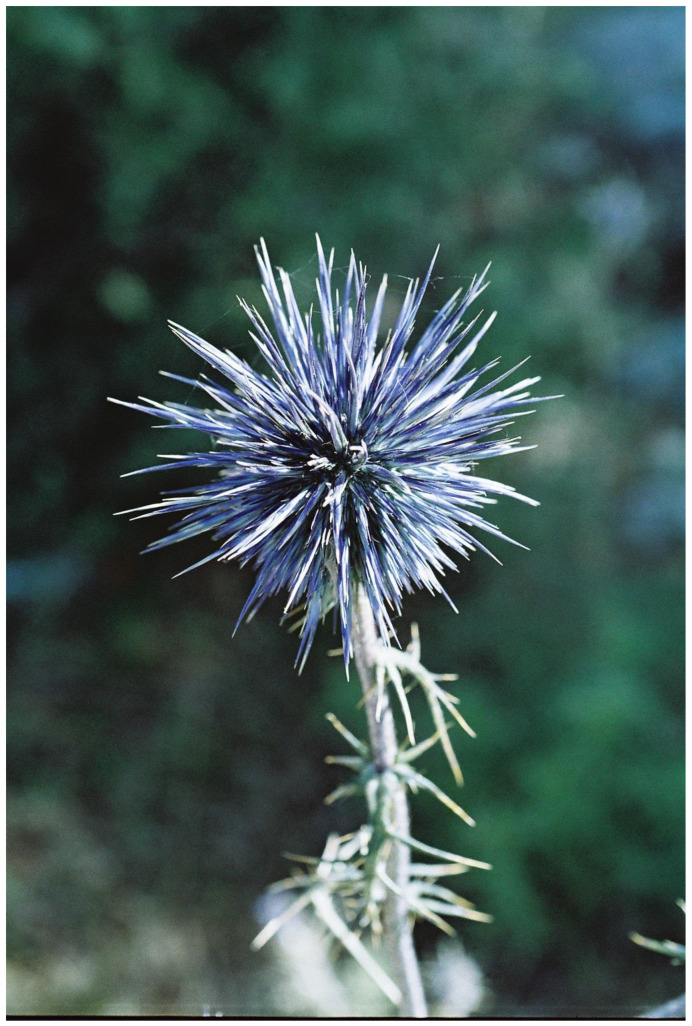
The bluish and highly spiny aposematic inflorescence of the Mediterranean species *Echinops adenocaulos* (Asteraceae), Mount Carmel, Israel.

**Figure 4:**
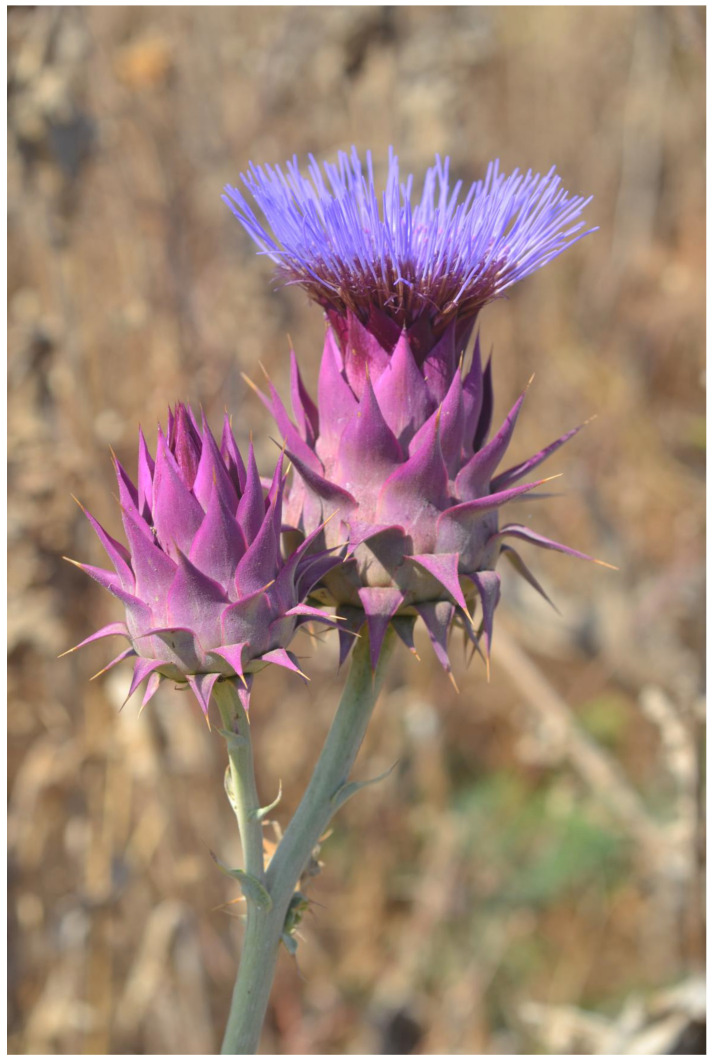
The red and spiny aposematic inflorescences of the Mediterranean species *Cynara syriaca* (Asteraceae) with their yellow spines, Jezrael Valley, Israel. The still young and closed inflorescence is as brightly colored as the flowering one. Both stages are very bitter. The combination of yellow spines and red bracts seems to serve not only pollinator attraction, but also mammalian herbivore repellence.

A very different type of flower aposematism is based on physical defense, i.e., by spines. My understanding that plants in general, and flowers in particular, may be visually aposematic emerged from my fieldwork in the Middle East. Here, many of the large herbivorous mammals that thrived since the Miocene and greatly influenced plant evolution, including defense, became extinct, especially in the last 15,000 years [109]. However, these extinct herbivores were replaced during the Holocene by large herds of sheep, goats, and cattle, which put huge grazing and browsing pressures on the wild vegetation for millennia. The fact that spines, thorns, and prickles are usually colorful as a global phenomenon [49] drew my attention to spines associated with flowers and inflorescences in the flora of Israel. It should be remembered that the Mediterranean climate differs from other climates with its wet winter and spring going hand in hand with green landscapes, and it has a very long and hot, rainless, and generally yellow summer [110,111]. Thus, plants that are green in the summer and flower then, and not in the lush and very green spring as is usually the case, may attract herbivores that look for water-containing and nutritious green plants. However, summer green plants are typically better defended against herbivory by being more toxic, spiny, or both than winter/spring green ones (see [112]). Focusing on the many spiny members of the Asteraceae, the spiniest plant family in the flora of Israel, it became clear that many of the spines associated with inflorescences are colorful (Figure 2, Figure 3 and Figure 4), although in the first years of the study [113], we still did not focus on spine and flower coloration but rather on morphology.

Then, we examined the seasonality of the flowering of spiny versus non-spiny species of Asteraceae, Fabaceae, and Lamiaceae, the three spiniest families in the Israeli flora. We found that the peak of flowering in the non-spiny species is in late March when the landscape is green, while the peak in the spiny ones is at the beginning of May, when the landscape is commonly dry and yellow [114]. At the level of the whole Israeli flora, we found that many of the spines associated with the flowers or inflorescences of annuals and herbaceous perennials are colorful; their colors are mainly yellow, red, orange, and white [55].

In order to further test the potential operation of flower aposematism, Lev-Yadun et al. [115] compared the distribution of the inflorescence colors of the 98 spiny versus the 189 non-spiny species of Asteraceae in the flora of Israel and found significant differences between the two groups. Yellow/white inflorescences dominate the non-spiny species that are commonly chemically defended, while pink/purple/blue flowers dominate the spiny species. Lev-Yadun et al. [115] hypothesized that the pink/purple/blue flowering of the spiny species may advertise the existence of deterring spines to mammalian herbivores. This putative aposematic signal is particularly conspicuous in the dry Mediterranean summer when the surrounding landscape turns yellow and the relative grazing pressure becomes much higher than that in the lush and green spring. This is in addition to the many colorful or otherwise conspicuous spines of these species [55,78].

Additional potential adaptive functions for pink/purple/blue flowering in summer-blooming species include increased visibility to pollinators and improved protection from radiation damage due to the flowers’ high anthocyanin content. The spine colors (in many Asteraceae species, the spines are yellow) differ significantly from the flower colors in the spiny species, suggesting that the spine and flower colors in many species may have evolved in response to different selective agents. The different colors of the flowers and spines may, however, just reflect the different cellular locations of pigments in the different cell types (vacuoles of parenchyma cells in flowers filled with red/purple/blue anthocyanins versus the membrane location of the hydrophobic yellow carotene-rich lignified hard cells of the spines) [115].

The strength of spine-based flower aposematism may be influenced by flower or inflorescence symmetry. This issue is discussed in the next section.

### 2.3. Flower Symmetry in Spiny and Toxic Plants and Aposematism

**Figure 5:**
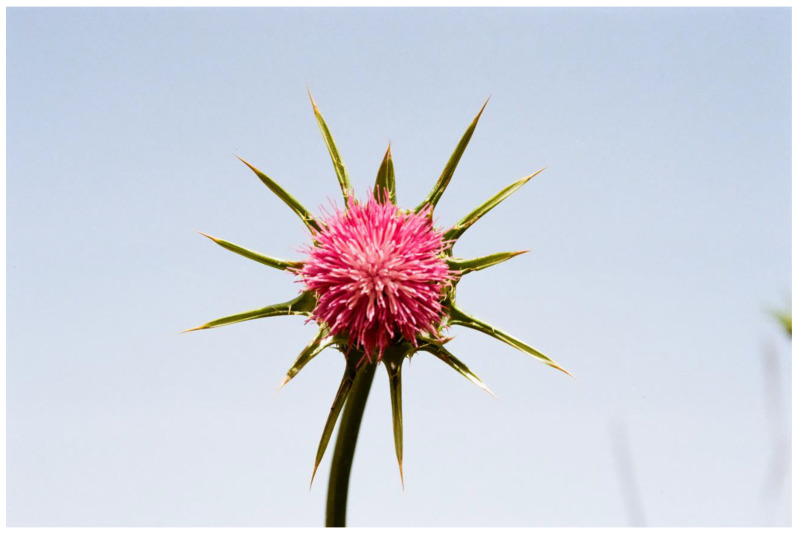
A typical symmetric, spiny, and aposematic inflorescence of *Silybum marianum* (Asteraceae), coastal plain, Israel.

**Figure 6:**
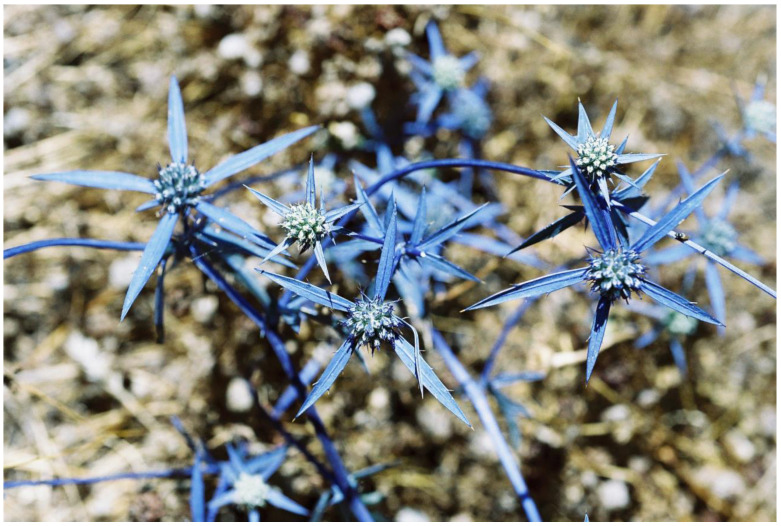
Symmetric blue leaf bracts with yellow spines of the visually aposematic flower heads of *Eryngium creticum* (Apiaceae), Mount Carmel, Israel.

**Figure 7:**
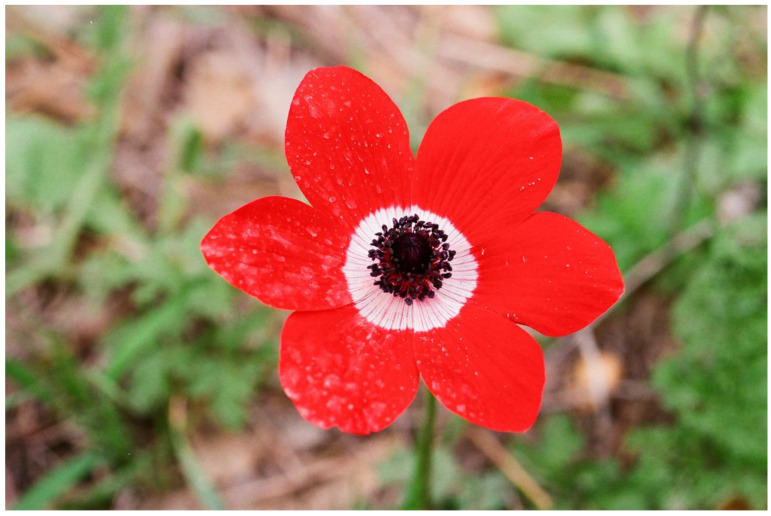
The symmetric and tri-colored (red, white, and black) toxic flowers that support the aposematism of *Anemone coronaria* (Ranunculaceae), Mount Carmel, Israel.

Symmetry is a basic characteristic of flowers and inflorescences [116,117,118,119]. It was proposed that symmetry increases the efficiency of visual aposematic animal displays [120,121,122]. Toxic colorful flowers, which were one of the first plant organs proposed to be aposematic [28,100], are, like almost all flowers, typically symmetric. Thus, Lev-Yadun [123] suggested that, as is the case in various animals, visual aposematism in spiny and poisonous plants also seems to be commonly associated with symmetry, including that of flowers and inflorescences. For instance, the flowering spiny inflorescences of many Near Eastern species of the Asteraceae (Figure 5), as well as those of other spiny taxa [55,113] (Figure 6), are radially symmetric. The same is true for the flowers of toxic taxa (Figure 1 and Figure 7).

Symmetry may stem from developmental constraints; in flowers, it may have other signaling purposes and may be involved in various pollinator manipulation tactics. There is an innate preference for symmetry in the visual systems of animals, probably because of the frequent need to recognize objects [124], which is a well-known fact from pollination biology [118]. Because of this, flowers may exploit this inherited mode of animal sensing that probably results in the animals paying more attention to symmetrical shapes and may serve both pollination and defense.

There is an obvious need for the experimental testing of the role of symmetry in visual plant aposematism. This is part of the general great need to test the hypotheses of plant aposematism, a subject that has received too little theoretical and experimental attention in comparison to animal aposematism [30,100,125,126].

### 2.4. Flower Aposematism by Volatiles

A dual olfactory action involving attracting pollinators while deterring other animals was found in various taxa (e.g., [127,128,129,130,131,132,133]). Thus, certain floral scents that commonly reflect flower and nectar chemistry may have a defensive role [11,130,131,133,134] in addition to their well-known attracting function (see below).

Two types of flower aposematism via volatiles were proposed. The first and more common one is the signaling of chemical toxicity. This type is discussed in this chapter. The second is the mimicry of animal or animal-related volatiles, which is discussed in a later chapter.

Pellmyr and Thien [135], in a broad theoretical study on the origin of angiosperms, proposed that attractive floral fragrances originated from chemicals serving as deterrents against herbivore feeding. In a much more focused study of flower defense in the genus *Dalechampia*, Armbruster [136] and Armbruster et al. [137] proposed that defensive resins had evolved into a pollinator reward system and that several defense systems had evolved from such advertisement systems. Pollen odors in certain wind-pollinated plants, which are not intended to attract pollinators, are rich in defensive molecules, such as α-methyl alcohols and ketones [138]. Herrera et al. [139] proposed that plants that possess a particular combination of traits that simultaneously enhance pollination and defend against herbivores enjoy a disproportionately higher fitness advantage over plants possessing only the individual traits of such combinations.

The dual action of attracting pollinators while deterring other animals was found in various taxa, e.g., *Catalpa speciosa* and *Aloe vryheidensis* [127,128,129]. Thus, in addition to their known pollinator-attracting function, floral scents may have a defensive role [11,133]. Junker and Blüthgen [130], in a meta-analysis of 18 previous studies, found that on average, the obligate flower visitors were usually pollinators, which were attracted to floral scent compounds, but facultative and mainly antagonistic flower visitors were strongly repelled by floral scents. They concluded that in general, benzenoids are attractive to pollinators, while monoterpenes, alcohols, ethers, and ketones are particularly repellent to facultative flower visitors. As usual in studies of flower signaling as repellence, aposematism was not mentioned.

### 2.5. Colorful, Toxic, and Bitter Nectars

The previous sections discussed aposematism by whole flowers or inflorescences. This section deals with deterrence by a specific small component of the reproductive organs, i.e., nectar.

Another aspect of apparent flower aposematism involves nectar. Nectar is the principal reward for pollinators [1,140]. However, nectar is produced not only in flowers but also in the leaves and branches of thousands of species (e.g., [141]), where it commonly attracts ants as bodyguards against herbivores (e.g., [141,142,143]). The patrolling defending ants may deter legitimate efficient pollinators, causing a conflict of interest for the plants [94,144,145]. Therefore, as described below, in order to overcome that conflict, the nectars and volatiles of many plant species deter ants from visiting the flowers.

Most nectars are colorless, but at least 68 species belonging to 20 genera in 15 families produce colored nectar (yellow, red, brown, black, green, and blue), and the main function of nectar color seems to be the attraction of vertebrate pollinators [129]. Some of the colored nectars are bitter, and it was proposed that in such cases the bad taste deters nectar robbers and mammalian herbivores [146,147], but not much evidence was found for the suggestion that it deters mammalian herbivores [129]. However, it was shown experimentally that the dark brown nectar of *Aloe vryheidensis* from South Africa, which is pollinated by various short-billed birds, repels non-pollinating honeybees and sunbirds (*Chalcomitra amethystine*) [128]. Aposematism was not discussed in the above cases, although visual or chemical signaling by plants to repel animals should be considered aposematism.

While typical floral nectar contains sugars and amino acids, many species also produce toxic compounds in nectar in addition to these nutrients [148,149,150]. Baker and Baker [151] suggested that certain nectars contain toxic or distasteful substances that deter nectar thieves. As what is toxic to one animal might be harmless to another [86,152], the nectar aposematism in a plant species, based on its chemotype, might be selective and not effective against all herbivores. A good case demonstrating this principle in relation to flowers, although not via nectar, is that of the Mediterranean geophyte *Anemone coronaria*. This toxic plant thrives and may even become dominant in heavily grazed habitats because most mammalian grazers do not eat it, but they do consume its non-toxic competitors [153]. However, roe deer (*Capreolus capreolus*) consume its nutritious flowers in large numbers [154]. The principle that what is toxic to one animal might be harmless to another is especially true for insects because many of them sequester the plant toxins for their own defense [155]. When toxic and bitter nectar is discussed, considering it as aposematism is not as straightforward as it is with flower color or odor because its deterrence potential is commonly dependent on tasting, i.e., repellence signaling by consumption and not by the usual signaling without consumption. There are probably many cases in which the toxicity is followed by olfactory signaling, but it is not easy to realize it because insects can sense volatile molecules at very low levels that humans do not sense. Moreover, measuring the volatiles in actual ecosystems is not easy, and in many cases, it is not accurate because many volatiles from other sources may occur in the air.

Tadmor-Melamed et al. [156] examined the influence of two pyridine alkaloids (nicotine and anabasine) in the nectar of *Nicotiana glauca* on the food consumption of its pollinator, the Palestine Sunbird (*Nectarinia osea*). They suggested that the deterrence by high concentrations of nectar alkaloids found in the nectar of certain individuals may lead the pollinating bird to visit more individual plants that express lower levels of nectar alkaloids, enabling it to be a more efficient pollinator. An elegant set of experiments using transgenic plants not expressing various volatiles showed that in the wild, *Nicotiana attenuate*’s volatile nicotine-repelled nectar thieves and, in a way, also pollinators. However, both repellent and attracting volatiles were found to contribute to seed production [157,158]. Barlow et al. [150] showed that the distasteful alkaloid-rich nectar of *Aconitum napellus* and *A*. *lycoctonum* was acceptable for their pollinator, the long-tongued bumblebee *Bombus hortorum*, but repelled the short-tongued nectar thief *B*. *terrestris*. Aposematism was not mentioned in these studies.

Janzen [159] suggested that ants do not approach the flower’s nectar in numerous plant species because that type of nectar contains chemicals that are unacceptable, indigestible, or toxic to ants. Later, in controlled experiments [127], ants that were given either a sugar solution or the toxic nectar of the tree *Catalpa speciosa* behaved differently. Those who were provided with the sugar solution consumed more, and they all descended the tree safely, whereas 27% of those who consumed the toxic nectar fell from the tree. Ants that defend the East African *Acacia* (*Vachellia*) *drepanolobium* and *A*. *zanzibarica* against herbivores are deterred from their flowers by an unknown volatile signal emitted by young flowers [160]. Floral repellents towards ants were shown in many other studies (e.g., [133,161,162,163,164,165]). All of the above studies demonstrated aposematism without mentioning it.

### 2.6. Flower Aposematism through Animal Mimicry

This section and the following one discuss flower defenses related to animal and animal action mimicry.

The mimicry of various animals, especially insects, is a well-known plant strategy for pollinator attraction (e.g., [6,53,84]). Of the many cases of such mimicry, the best-known case is that of bee-mimicking orchids. It is probable that in this case the simultaneous pollinator attraction and herbivore repellence is also involved.

The repelling of bees by the bee-mimicking *Ophrys* flowers was proposed in the year 1831, almost 200 years ago, by Robert Brown, one of the greatest botanists of all time (of the Brownian movement, who discovered that plant cells have a nucleus, along with many other important discoveries in plant biology). Despite significant efforts to find his text on the web, I could not find the original text because it was cited several times without the reference being given. Darwin ([166], pp. 55–56), with regard to the pollination of the bee orchid *Ophrys*, wrote “Robert Brown imagined that the flowers resembled bees in order to deter their visits, but this seems extremely improbable. The flowers with their pink sepals do not resemble any British bee”. Darwin did not give a reference or date to the above citation. Ames and Ames [167], without giving the reference, commented “Robert Brown was of the opinion that the flowers of *Ophrys apifera* resemble bees to repel, not to attract”. Wickler [53], in his classic book about mimicry, mentioned, again without giving the reference, that Brown in the year 1831 expressed the opinion that *Ophrys* species scare off insects with their bee-mimicking flowers. Thus, it seems that visual aposematism by *Ophrys* flowers towards bees or herbivores was considered 60 years before the term “aposematism” was coined by Poulton [168] with regard to defensive visual signaling by defended animals towards predators and 30 years before aposematism (before coining the term) and mimicry by the defended (Müllerian) and the non-defended (Batesian) were demonstrated in butterflies of the Amazon [74].

**Figure 8:**
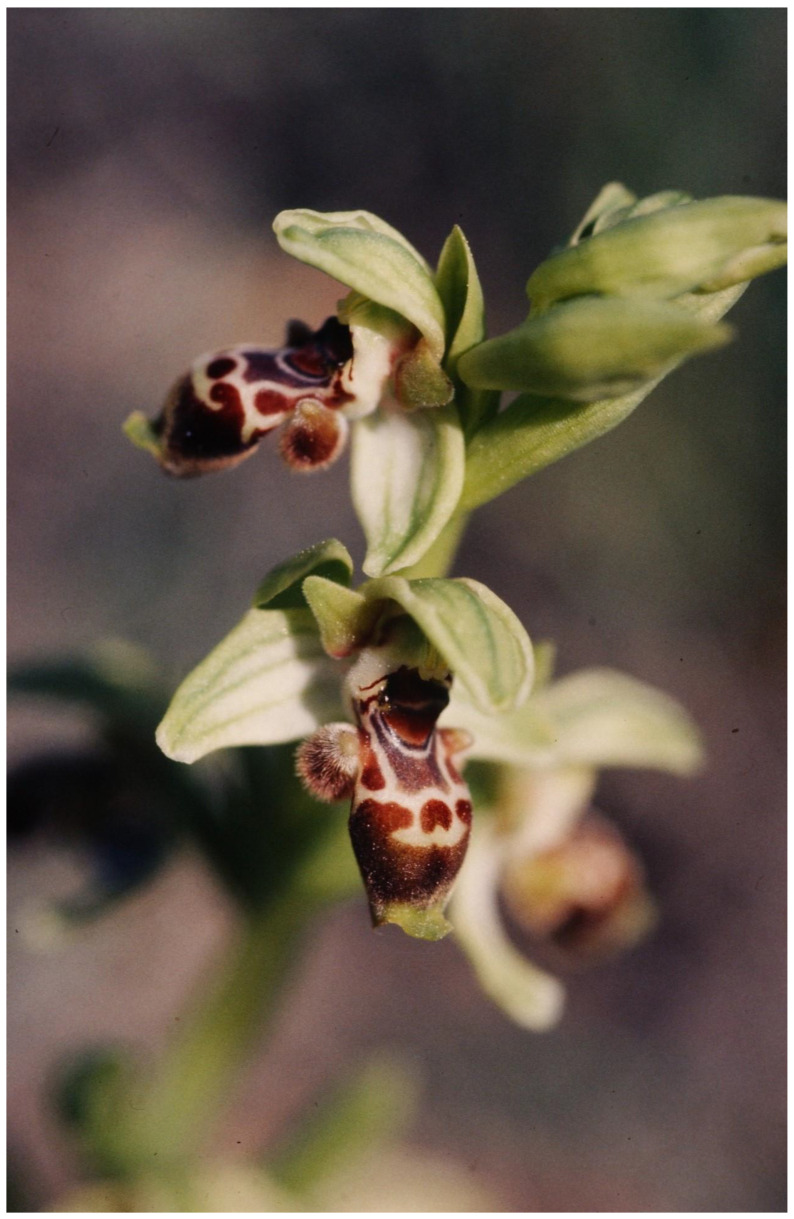
Visual bee mimicry by *Ophrys umbilicate* (Orchidaceae), a geophyte common in heavily grazed habitats in the Levant, Mount Carmel, Israel.

Lev-Yadun and Ne’eman [169] proposed re-considering and experimentally testing the century-old almost-forgotten anti-herbivory role of visual bee mimicry that was discussed by Rolfe [170] with regard to a comment by Mr. E. Kay Robinson, in a letter to the British newspaper “Daily News” (founded by Charles Dickens). Mr. E. Kay Robinson proposed in that letter that bee mimicry by various flowers was not aimed at pollinator attraction, but rather at deterring grazing cows [cited in 170]. While Rolfe [170] dismissed the anti-herbivory hypothesis, which was later practically forgotten, Lev-Yadun and Ne’eman [169] proposed that it may also be a part of the explanation for the visual bee or wasp mimicry by orchid flowers, especially considering that olfactory mimicry is the dominant species-specific pollinator attractor. Lev-Yadun and Ne’eman [169] proposed that many large (mostly mammalian) herbivores and some herbivorous insects may be deterred by visual bee or wasp mimicry, and that defensive mimicry of the bee pheromone in *Ophrys* flowers (Figure 8) should also be considered in this context, especially in deterring herbivorous insects that can potentially sense the volatiles. Lev-Yadun and Ne’eman [169] suggested that this visual anti-herbivory bee or wasp mimicry was a case of Batesian mimicry, i.e., deceptive aposematism.

Lev-Yadun and Ne’eman [169] suggested that this defensive mimicry was not exclusive and that it probably played a secondary role in pollination. They extended this hypothesis to many rewarding flowers of other taxa that are bee- or wasp-pollinated and proposed that an abundance of pollinating bees or wasps may deter herbivorous mammals and insects from the flowers during the most sensitive period of their peak flowering season.

**Figure 9:**
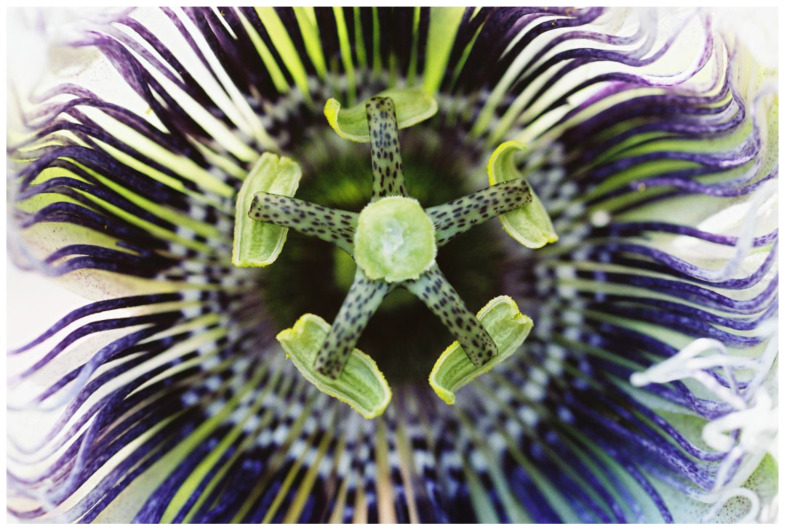
Potential ant mimicry in the flower of *Passiflora* sp., grown as an ornamental in the central coastal plain, Israel, by coloration in the shape of many small dark dots and short lines.

Ants are fierce defenders of their host plants against herbivores; therefore, thousands of plant species provide ants with nectar, food bodies, and cavities for housing [171]. Lev-Yadun [172] suggested that visual ant mimicry in the flowers of many *Passiflora* species in the shape of numerous dark spots (Figure 9) may serve in repelling insects from laying eggs there; this is similar to the defensive butterfly egg mimicry common in *Passiflora* leaves ([173] and citations therein).

**Figure 10:**
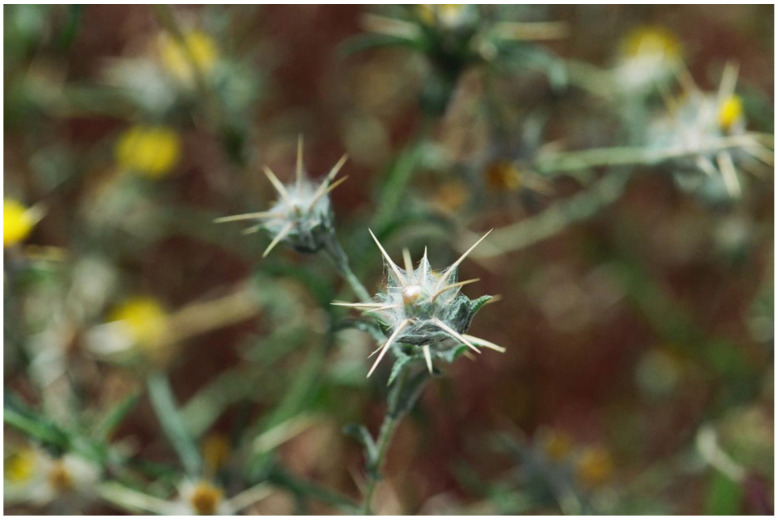
Spider web mimicry by the inflorescence of *Centaurea* sp., Mount Adir, Upper Galilee, Israel.

Yamazaki and Lev-Yadun [174] suggested that there are many cases where dense white trichomes that cover plant surfaces look like spider webs; due to this covering, various herbivores are repelled. This is the case, for instance, in the inflorescences of various members of Asteraceae, e.g., *Carthamus lanatus*, *Centaurea melitensis*, and other *Centaurea* species (Figure 10); many very young inflorescences of the hemicryptophyte *Gundelia tournefortii*; and several members of the genus *Onopordum*, as well as *Arctium tomentosum*. This is a type of visual inflorescence aposematism, although Yamazaki and Lev-Yadun [174] did not consider it to be aposematism.

A very different type of repellence by animal mimicry was discovered in the flowers of pyrethrum (*Tanacetum cinerariifolium*, Asteraceae). This plant repels herbivores and attracts carnivores by producing the aphid alarm pheromone (*E*)-β-Farnesene. Ladybug beetles that consume aphids are attracted by the pheromone, and the aphids are repelled [175]. The use of the aphid alarm pheromone is not restricted to flowers; it was also found in the leaves of wild potatoes four decades ago [176].

I suggest that all of the above are cases of either visual or olfactory aposematism.

### 2.7. Flower Aposematism through Carrion or Dung Odor Mimicry

**Figure 11:**
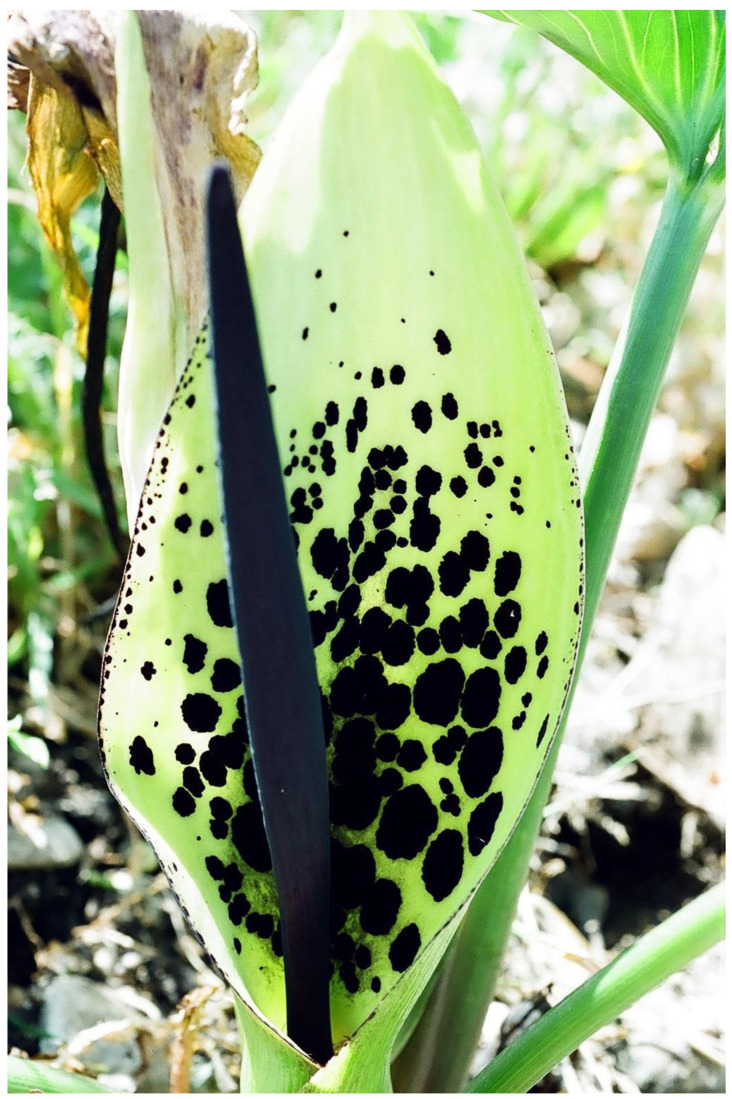
The dung-smelling inflorescence of *Arum dioscoridis* (Araceae), Mount Carmel, Israel.

Many flower species belonging to many families found on all continents attract pollinating insects by mimicking the odors of brood sites, especially of carrion, dung, or rotting organic materials. This characteristic is best known in the Araceae family (Figure 11) and in stapeliads (e.g., [1,6,84,134,177,178,179]) and is based on sensory exploitation [178,180].

Lev-Yadun et al. [70] suggested that these odors may repel mammalian herbivores at the same time that they attract pollinators because carrion odor is a good predictor (a cue) for two potential dangers to mammalian herbivores: (1) pathogenic microbes and (2) the proximity of carnivores. Dung odor predicts (cues about) feces-contaminated habitats that present high risks of parasitism. The refraining from feces-contaminated grass by mammalian herbivores is well known (see [50]). Carrion odors have a high probability of a carcass being defended by a predator because all predators will store or consume carrion. These carrion and dung odors, which for decades were considered only as attractants of pollinators, were thus proposed by Lev-Yadun et al. [70] to be two new types of repulsive olfactory aposematic mimicry by flowers: (1) olfactory feigning of carcass, a well-known behavioral defensive strategy (thanatosis) in animals [44,48] and (2) olfactory mimicry of feces, which also has a defensive visual parallel in animals [51,181] and probably also in plants [30,88]. In sum, the above unusual cases are types of olfactory aposematism.

## 3. Conclusions and Further Research

This short essay illustrates the scattered known facts about flower aposematism and also aims to stimulate further targeted research on flower aposematism. In sum, despite the huge body of research conducted to characterize visual and chemical signaling by plants towards pollinating animals, the aposematic hypothesis for the parallel defensive signaling by this very important plant organ, i.e., the flower, which is often visually and chemically conspicuous, has received very little attention. The data accumulated following decades of studying floral biology without considering the aposematic hypothesis clearly show that many plant species belonging to many taxa and found in many types of ecologies repel herbivores (including nectar thieves), inefficient pollinators, and even mutualistic bodyguards such as ants from their flowers. The above is actual flower aposematism, and it seems to be quite common. Both visual and chemical flower aposematism deserve recognition and target-oriented research. However, without a broad recognition that aposematism is commonly expressed by flowers, the future progress in understanding it will continue to be very slow.

Further research on flower aposematism should integrate plant biology with pollinator and herbivore biology. Studying flower aposematism in depth requires the collaboration of zoologists with plant biologists and the use of many of the wonderful molecular tools of current biology (e.g., [152]) in order to understand its biology and evolution, as was conducted, for instance, in the related subject of insect mimicry in order to attract pollinators to the flowers of *Gorteria diffusa* [182].

## Data Availability

The data are contained within the article.
